# Abnormal expression of GADD45B in human colorectal carcinoma

**DOI:** 10.1186/1479-5876-10-215

**Published:** 2012-10-30

**Authors:** Lisha Wang, Xiuying Xiao, Dali Li, Yayun Chi, Ping Wei, Yiqin Wang, Shujuan Ni, Cong Tan, Xiaoyan Zhou, Xiang Du

**Affiliations:** 1 Department of Pathology, Fudan University Shanghai Cancer Center, Shanghai, 200032, China; 2 Department of Oncology, Shanghai Medical College, Fudan University, Shanghai, 200032, China; 3 Institute of Pathology, Fudan University, Shanghai, 200032, China; 4 Institutes of Biomedical Sciences, Fudan University, Shanghai, 200032, China; 5 Department of Oncology, Shanghai Xuhui District Center Hospital, Shanghai, 200031, China; 6 Cancer Research Institute, Fudan University Shanghai Cancer Center, Shanghai, 200032, China

**Keywords:** Colorectal carcinoma, GADD45B, Carcinogenesis, Relapse

## Abstract

**Background:**

GADD45B is a member of the growth arrest DNA damage-inducible gene family associated with cell growth control, apoptosis, and DNA damage repair response. The aim of this study is to detect the role of GADD45B in colorectal carcinoma (CRC); the area not studied in depth to date.

**Methods:**

The mRNA and protein levels of GADD45B were examined by Real-Time quantitative PCR (RT-qPCR) and immunohistochemistry (IHC) in CRC tissues and adjacent noncancerous tissues (ANCT). Over-expression plasmids and SiRNA were used to regulate GADD45B expression in CRC cell lines in vitro and flow cytometry and Western blotting were used to detect apoptotic changes.

**Results:**

The mRNA and protein levels of GADD45B were significantly higher in CRC tissues than those in ANCT (*P*<0.05). Up-regulation of GADD45B was also correlated with relapse and death of CRC patients (*P*<0.05). The Kaplan-Meier survival curves indicated that disease-free survival (DFS) was significantly worse in CRC patients who showed GADD45B overexpression. A Cox multivariate analysis revealed that GADD45B overexpression and TNM stage were significant factors affecting patients’ survival. On the other hand, as a tumor suppressor gene, GADD45B amplified from normal colorectal tissues could induce apoptosis in CRC cell lines and may be associated with the p53-mediated apoptotic pathways.

**Conclusion:**

GADD45B, a tumor suppressor gene potentially through the p53-mediated apoptotic pathways, is paradoxically overexpressed in CRC and as such may play an unappreciated role in tumorigenesis. The exact mechanism of GADD45B inactivation and overexpression requires further investigation. GADD45B could be a potential therapeutic target for CRC treatment in future.

## Background

CRC is one of the most common malignancies worldwide [1]. Many risk factors, such as inflammatory bowel disease, inherited syndrome, diets low in fiber, smoke, obesity, and heavy alcohol use, are related to intestinal mucosa injury and dysplasia, and may result in carcinogenesis of CRC [2,3]. However, the potential molecular mechanisms of DNA damage from these exposures are unknown. The development of CRC is a long and complicated process, including the activation of multiple oncogenes and the inactivation of tumor suppressor genes.

GADD45B is a member of the growth arrest DNA damage-inducible gene (GADD45) family, which is composed of GADD45A (GADD45), GADD45B (MYD118), and GADD45G (cytokine response gene 6). This gene family encodes small (18 kDa), evolutionarily conserved proteins, sharing homology and high acidity [4]. GADD45B was previously identified as a primary response gene in myeloid differentiation activated by interleukin-6 in the mouse myeloid leukemia cell line M1, which is associated with cell growth control, apoptosis, and cellular responses to DNA damage [5]. Recent studies have reported that GADD45B may play an important role in the carcinogenesis of human hepatocellular carcinoma and pituitary gonadotrope tumors [6-8], but its role in CRC remains unclear. Therefore, we examined both the mRNA and protein expression of GADD45B, correlated the results with clinicopathologic characteristics of CRC patients, and detected GADD45B-induced apoptosis in CRC cell lines.

## Methods

### Patient samples

RNAlater-preserved solid tissues, formalin-fixed paraffin-embedded (FFPE) archival CRC tissues and ANCT were obtained from Fudan University Shanghai Cancer Center between 2005 and 2008. The tumors were assessed according to the WHO classification by two academic gastrointestinal pathologists. The inclusion criteria were as follows: colorectal adenocarcinoma (excluding mucinous carcinoma); no preoperative chemotherapy or radiotherapy; primary sporadic tumors. This study was approved by the Ethical Committee of Fudan University Shanghai Cancer Center for Clinical Research. The written informed consents were obtained from all the patients.

### Cell culture and reagents

Dulbecco’s Modified Eagle’s Medium (DMEM), Roswell Park Memorial Institute (RPMI) 1640 medium, penicillin, streptomycin, Fetal Bovine Serum (FBS), trypsin-EDTA (Ethylene Diamine Tetraacetic Acid) and Phosphate Buffered Saline (PBS) were purchased from GIBCO BRL (USA). Three human CRC cell lines, including LoVo, SW620, and SW480 were purchased from American Type Culture Collection (ATCC). LoVo (poorly differentiated) and SW620 (highly metastatic) were cultured in high glucose DMEM with 10% FBS, 100 units/ml penicillin and 100 μg/ml streptomycin. SW480 (highly differentiated) was cultured in RPMI 1640 supplemented with 10% FBS, 100 units/ml penicillin, and 100 μg/ml streptomycin. All cells were cultured in a 5% CO_2_ incubator at 37°C. The primary antibodies GADD45B (H-70), P53 (DO-1), Bcl-2 (100), Bax (N-20), and caspase-3 p20 (N-19) were purchased from Santa Cruz Biotechnology; β-actin (13E5), cleaved PARP (Asp 214) and the secondary antibodies (horseradish peroxidase–linked anti-mouse immunoglobulin G, and anti-rabbit immunoglobulin G) were purchased from Cell Signaling Technology.

### RNA extraction and quantitative analysis of GADD45B

Total RNA was extracted from 64 pairs of CRC tissues, ANCT, and cultured CRC cells using TRIzol reagent (Invitrogen, Carlsbad, CA, USA) following the manufacturer’s instructions. The structural integrity of the total RNA was confirmed by electrophoresis on 1% agarose gels. The first strand of cDNA was reverse-transcribed from 500 ng total RNA in 20 μl using the cDNA Synthesis Kit (Tiangen Biotech Co., Ltd., Beijing). GAPDH was used as an endogenous control. The cycling conditions for GAPDH and GADD45B were as follows: one cycle of 95°C for 5 minutes; 40 cycles of 95°C for 20 seconds, 58°C for 30 seconds, and 68°C for 45 seconds; and one cycle of 72°C for 10 minutes. The specificity of amplification was validated by a single peak in the melting curves. The GADD45B gene was amplified with the following primes: 5^′^-TGA CAA CGA CAT CAA CAT C-3^′^ (the forward primer) and 5^′^-GTG ACC AGA GAC AAT GCA G-3^′^ (the reverse primer). The endogenous GAPDH gene was amplified with the following primers: 5^′^-GAA AGT CCG GAA GTC TCT GG-3^′^ (the forward primer) and 5^′^-TAG AGA CTT GGG CAG TGT GG-3^′^ (the reverse primer). The standard curves for GAPDH were generated using serial dilutions. Each RT-qPCR cycle was repeated three times to confirm the results.

### IHC analysis

152 FFPE blocks of CRC tissues and ANCT were collected for tissue microarrays. Two CRC tissue cores and two ANCT cores from the same patient’s FFPE blocks were arranged on a recipient paraffin block (with a 1 mm core per specimen). 5-μm thick paraffin sections were deparaffinized in xylene and rehydrated in a graded alcohol series, boiled with 10 mmol/L of citrate buffer (pH 6) for 15 min, treated with 0.3% H_2_O_2_ for 10 min, and preincubated in blocking solution (10% normal goat sera) for 1 h at room temperature. The steps were performed using the Envision two-step method. The Envision and DAB Color Kit was purchased from Gene Tech (Shanghai) Company Limited. The GADD45B rabbit anti-human polyclonal antibody was used at a 1:500 dilution. PBS was used as a negative control.

Tissue microarray slides were concurrently evaluated by 2 of the authors. A granular cytoplasmic stain was assessed as positive. Briefly, the staining index (SI, range 0–9) was considered as the product of the intensity score (0, no staining; 1+, faint/equivocal; 2+, moderate; 3+, strong) and the distribution score (0, no staining; 1+, staining of <10% of cells; 2+, between 10% and 50% of cells; and 3+, staining of >50% of cells). For GADD45B protein in this study, a moderate/strong cytoplasm staining of (SI = 3–9) was defined as positive staining, while a weak or negative staining (SI = 0–2) was defined as negative staining [9,10].

### Construction of pEGFP-N1-GADD45B

cDNA from normal colorectal tissues was amplified by PCR. The PCR product and the pEGFP-N1 plasmid vector were double digested with BglII/SalI enzymes and then ligated using T4 DNA ligase. The ligated product was transformed into *Escherichia coli* DH5α competent cells, coated to the LB solid medium containing kanamycin (100 ug/ml), and stored at 37°C overnight. A selection of white colonies was amplified using the PCR reaction, and the positive clone was incubated in a LB liquid medium (kanamycin 100 ug/ml) with agitation overnight. The recombinant plasmid was extracted the next day. Identification of the correct plasmid was sent to Shanghai R&S Biotechnology for sequencing. The result of DNA sequencing was consistent with the PubMed database. The plasmid sequence was named pEGFP-N1-GADD45B. SW480 (3*10^5^ cells/dish) were transplanted into 35 mm dishes 1 day before transfection. After 70% confluence was reached, 2 μg of plasmid DNA was diluted with 100 μl serum-free medium (without antibiotics). The transfection complex was then formed by adding the FuGENE® HD Transfection Reagent (Roche Diagnostics, Mannheim, Germany) to the tube containing the diluted DNA. The transfection complex was mixed and incubated for 15 minutes at room temperature. The mixture was then added to each dish (there is no need to remove the old medium and replace it with fresh medium) and stored in a 5% CO_2_ incubator at 37°C.

### SiRNA synthesis

Small interfering RNA (SiRNA) duplexes were synthesized and tested for the specific inhibition of GADD45B expression. SW620 (3*10^5^ cells/dish) were transplanted into 35 mm dishes 1 day before transfection. After 70% confluence was achieved, 100 pmol Si-GADD45B (purchased from Shanghai GenePharma Co., Ltd.) was diluted with 100 μl serum-free medium using the FuGENE® HD Transfection Reagent, according to the manufacturer’s instructions.

### Cell apoptosis analysis

The induction of apoptosis by Si-GADD45B and pEGFP-N1-GADD45B in SW620 and SW480 was determined by flow cytometry using the Annexin V-FITC Apoptosis Detection Kit. Briefly, 3*10^5^ cells were plated and treated with Si-GADD45B and pEGFP-N1-GADD45B for 24 hours. The cells were then harvested, washed in PBS, and incubated with Annexin V and propidium iodide in a binding buffer in the dark at room temperature for 10 minutes. The stained cells were analyzed using the Beckman Coulter Flow Cytometer.

### Western blotting analysis

Whole cell lysates were generated using the cell lysis solution, followed by centrifugation and the collection of the supernatant fraction for immunoblotting. Proteins were separated using SDS-PAGE gel electrophoresis and transferred onto a nitrocellulose membrane. After blocking with 5% non-fat milk in blocking buffer (20 mmol/l of PBS containing 0.1% Tween 20), the membrane was incubated with the primary antibody at 4°C overnight and incubated with the appropriate horseradish peroxidase-conjugated secondary antibodies the next day for 1 hour at room temperature. The immunoreactive bands were visualized using the ECL Plus Western Blotting Detection System. The level of β-actin for each sample was used as a loading control.

### Statistical analysis

The experimental data were expressed as the mean ± standard deviation, and the statistical significance between different groups was determined using t-tests. The relationship between GADD45B expression and clinicopathological features of CRC patients was analyzed using χ2 and Fisher’s exact tests. Statistical significance was defined as a *P*-value less than 0.05.

## Results

### Quantitative analysis of GADD45B mRNA expression

GADD45B and GAPDH mRNA expression was calculated using the absolute quantification method according to the parameter threshold cycle (Ct) and gene copy numbers [11]. Melting curves of GADD45B and GAPDH with single peaks indicated good specificity. The standard curve formulas, Y=35.9–4.555X (r^2^=0.999), for GAPDH was derived from the lines of the calibration curves. The average amount of GADD45B mRNA in CRC tissues was significantly higher than that in ANCT (*P* <0.05, Table 1).

**Table 1:** GADD45B expression in CRC tissues and ANCT

	**n**	**GADD45B mRNA**	***P***	**n**	**GADD45B protein**		**χ**^**2**^	***P***
					**Positive**	**Negative**		
CRC	64	23625.6±4028.1^a^	<0.05	152	105 (69.1%)	47 (30.9%)	45.805	0.000
ANCT	64	21318.2±4549.9		152	46 (30.3%)	106 (69.7%)		

^a^ gene copy numbers using SYBR Green.

### IHC analysis of GADD45B protein expression

**Figure 1:**
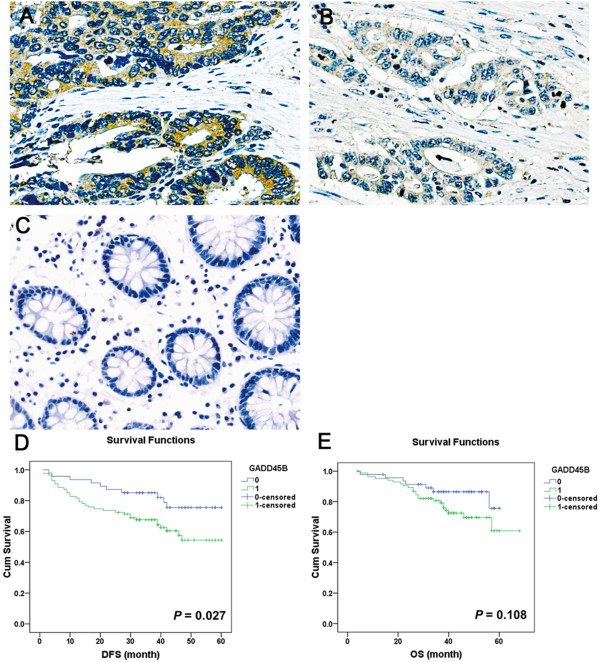
**Illustrates IHC of GADD45B in tissue microarrays (EnVision×400).** (**A**) CRC tissues showing strong cytoplasmic staining. (**B**) CRC tissues showing little or very weak staining of epithelia. (**C**) ANCT showing negative staining. (**D**-**E**) Relationship between GADD45B expression and DFS/OS. 0: GADD45B not overexpressed; 1: GADD45B overexpressed.

In the IHC analysis, positive staining of GADD45B protein was noted in 109 of the 152 (69.1%) paraffin-embedded CRC tissues, while negative staining was noted in the remaining cases (30.3%, 46 of 152, *P*<0.05) (Table 1, Figure 1). Table 2 summarizes the correlation of GADD45B expression with the clinicopathological features of CRC patients. The age of patients ranged from 29 to 75 years with a median age of 63 years; 54.6% were men (83 of 152), and 45.4% were women (69 of 152). GADD45B expression was not correlated with patients’ sex, age, tumor size, location, gross type, differentiation and TNM stage. The up-regulation of GADD45B was correlated with the relapse and status (survival or death) of CRC in patients (*P*<0.05). Patients with higher GADD45B expression tended to have a higher risk of relapse and death.

**Table 2:** Relationship between GADD45B expression and clinicopathological features in CRC patients

**Clinicopathological features**	**n**	**GADD45B n (%)**	**χ2**	***P***
Gender			0.116	0.734
Male	83	59 (71.1%)		
Female	69	46 (66.7%)		
Age (years)			0.400	0.527
<60	93	66 (71.0%)		
>=60	59	39 (66.1%)		
Tumor size (cm)			0.079	0.779
<5	88	60 (68.2%)		
>=5	64	45 (70.3%)		
Location			3.143	0.076
Colon	101	65 (64.4%)		
Rectum	51	40 (78.4%)		
Gross type			0.055	0.973
Massive	57	42 (70.0%)		
Ulcerative	81	57 (68.7%)		
Invasive	8	6 (66.7%)		
Differentiation			2.280	0.320
Well	5	3 (60%)		
Moderate	113	75 (66.4%)		
Poor	34	27 (79.4%)		
TNM stage			2.114	0.146
II	87	56 (64.4%)		
III	65	49 (75.4%)		
Relapse			5.390	**0.020**
Yes	47	37 (78.7%)		
No	90	53 (58.9%)		
Status			4.269	**0.039**
Survival	102	62 (60.8%)		
Death	35	28 (80.0%)		

All patients were followed up for at least 4 years. After 4 years, 102 patients were still alive, 35 patients succumbed to the disease, and 15 patients declined to continue the participation in the study. The median survival time of CRC patients was 48 months. DFS was significantly worse in CRC patients with GADD45B overexpression than in patients that did not overexpress GADD45B (*P*= 0.027, Figure 1D, 1E, Table 3). As shown in Table 3, stage Ш patients were also significantly associated with a poorer DFS (*P* = 0.013) compared with stage II patients. To assess whether GADD45B and TNM stage could be used as independent prognostic factors or not, a Cox model was generated using univariate analysis. The results indicated that combined analysis of GADD45B and TNM stage was of statistically significance (χ^2^=9.979, *P*=0.007).

**Table 3:** Univariate regression model of prognostic covariates in CRC patients

**Characteristics**	**DFS**		**OS**	
	**χ2**	***P***	**χ2**	***P***
Sex	0.835	0.361	0.973	0.324
Age	0.104	0.747	1.716	0.190
Location	0.160	0.689	0.017	0.897
Size	0.020	0.888	0.207	0.649
Gross type	0.832	0.660	0.322	0.851
Differentiation	3.939	0.140	1.972	0.373
TNM	6.155	**0.013**	1.793	0.181
GADD45B expression	4.915	**0.027**	2.586	0.108

Abbreviations: *DFS* disease free survival, *OS* overall survival.

The results represent poorer DFS in stage III patients and GADD45B-overexpressed patients.

### Detection of GADD45B background expression in CRC cell lines

**Figure 2:**
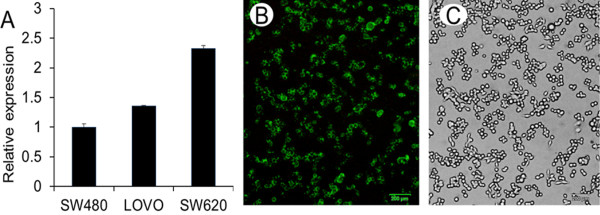
**Displays GADD45B expression in CRC cell lines and transfection efficiency.** (**A**) GADD45B expression was different in three CRC cell lines: highest in SW620, lowest in SW480, and moderate in LoVo. (**B**-**C**) Fluorescent (GFP) image and bright field image (the same area) of SW620 at 24 h after transfection under converted fluorescent microscopy.

RT-qPCR was used to qualify GADD45B expression in different CRC cell lines, including LoVo, SW480, and SW620. As shown in Figure 2A, mRNA expression of GADD45B was the highest in SW620, the lowest in SW480, and moderate in LoVo. Therefore, in the following work, we chose SW480 for overexpression experiments and SW620 for inference experiments.

### Successful construction of pEGFP-N1-GADD45B and synthesis of Si-GADD45B

To determine whether the construction of pEGFP-N1-GADD45B was successful, the recombinant plasmid was verified using PCR and electrophoresis. In the 483 bp location, there were evident bands of the PCR products. Meanwhile, the pEGFP-N1-GADD45B plasmid structure was also confirmed by double-enzyme cleavage (BgII/SaI enzyme) and gene sequence analysis. Two aim bands, at 4.7 kb and 483 bp, were acquired. After the successful construction of pEGFP-N1-GADD45B and the chemical synthesis of the Si-GADD45B duplexes, plasmid DNA and Si-RNA were transfected into CRC cell lines. The transfection efficiency was between 70% and 80%, as assessed with converted fluorescent microscopy (Figure 2B, 2C).

### Apoptosis analysis in human CRC cells

**Figure 3:**
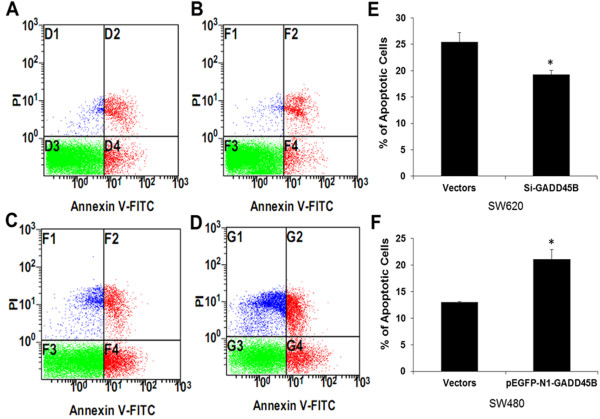
**Shows induction of apoptosis in human CRC cell lines.** (**A**) Apoptosis assay in SW620 transfected with vectors as control. (**B**) Apoptosis assay in SW620 transfected with Si-GADD45B. (**C**) Apoptosis assay in SW480 transfected with vectors as control. (**D**) Apoptosis assay in SW480 transfected with pEGFP-N1-GADD45B. (**E**-**F**) Apoptotic changes in SW620 and SW480 after transfection. * indicates *P*<0.05.

To determine whether the overexpression or inhibition of GADD45B in CRC cells was associated with the induction of apoptosis, SW480 and SW620 cells were treated with pEGFP-N1-GADD45B and Si-GADD45B as described above. The numbers of apoptotic cells were assessed using the Annexin V-FITC Apoptosis Detection kit. Transfection with Si-GADD45B in SW620 for 24 hours resulted in a significant decrease of apoptotic cells (Figure 3A, 3B); while transfection with pEGFP-N1-GADD45B in SW480 for 24 hours resulted in a significant increase of apoptotic cells (Figure 3C, 3D). This indicated that pEGFP-N1-GADD45B could effectively induce apoptosis in CRC cell line, while Si-GADD45B had the opposite effect (*P*<0.05, Figure 3E, 3F).

### Expression of apoptosis-related proteins by Western blotting

**Figure 4:**
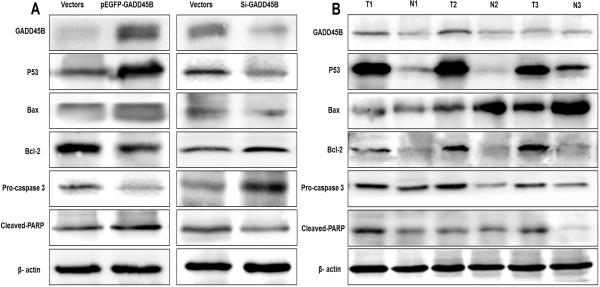
**Confirms expression of GADD45B and apoptosis-related proteins by Western blotting.** (**A**) Expression of GADD45B and apoptosis-related proteins in CRC cell lines after transfection with pEGFP-N1-GADD45B and Si-GADD45B. (**B**) Expression of GADD45B and apoptosis-related proteins in CRC tissues and ANCT.

Transfection of CRC cells with pEGFP-N1-GADD45B resulted in an increase in GADD45B, while transfection with Si-GADD45B showed a decrease in GADD45B (Figure 4A). Early studies showed that p53 can bind the Bax gene promoter region and regulate Bax gene transcription [12,13]. The Bcl-2 protein family plays a critical role in the regulation of apoptosis [14]. Because we observed apoptotic changes in CRC cells, we analyzed the levels of P53, Bax and Bcl-2 in cells treated with pEGFP-N1-GADD45B and Si-GADD45B, respectively. SW480 transfected with pEGFP-N1-GADD45B showed an increase in the level of P53 and Bax protein, with a concomitant decrease in the level of Bcl-2, when compared with the control cells. SW620 transfected with Si-GADD45B showed a decrease in the level of P53 and Bax protein, with a concomitant increase in the level of Bcl-2 (Figure 4A).

Caspase-3, as a critical executioner of apoptosis, cleaves a broad spectrum of cellular target proteins, including nuclear PARP that leads to a cell death cascade [15]. To clarify how the apoptotic pathway was activated by GADD45B, we examined the effects of pEGFP-N1-GADD45B on the activation of Caspase-3 and PARP. Transfection with pEGFP-N1-GADD45B in SW480 resulted in an obvious increase in the cleavage of PARP and a decrease in unactivated caspase-3 (Figure 4A). This indicates that the canonical mitochondrial apoptotic pathway may be activated by pEGFP-N1-GADD45B.

In addition, expression of these apoptosis-related proteins was also detected in CRC tissues. In paired CRC tissues and ANCT, apoptosis-related proteins expressed differently (Figure 4B). P53 and Caspase-3 has the similar change trend with GADD45B, indicating similar implications for prognosis.

## Discussion

CRC carcinogenesis is a complicated and multifactorial process resulting from many environmental exposures. This process involves the combined actions of multiple oncogenes and tumor suppressor genes [16]. As a negative growth-control gene, GADD45B is implicated in DNA damage, cell cycle arrest, and apoptosis. According to reports in the literature, higher expressions of GADD45B are associated with a higher risk of recurrence based on a cluster analysis of patients with stage II/III colon cancer treated with surgery alone or surgery plus adjuvant fluorouracil plus leucovorin [17]. Our study was consistent with the above research. To investigate the role of GADD45B in CRC, we first detected GADD45B expression in 64 pairs of CRC and ANCT by RT-qPCR. The results indicated that GADD45B was significantly up-regulated in CRC. Tissue microarrays are reliable tools for the clinicopathological characterization of cancer tissues [18,19]. The IHC analysis in 152 CRC patients using tissue microarrays further confirmed the results of the RT-qPCR and also observed that up-regulation of GADD45B expression was correlated with relapse and death in CRC patients (*P*<0.05). The patients with higher GADD45B expression tended to have a higher risk of relapse and death. The Kaplan-Meier survival curves indicated that DFS was significantly worse in CRC patients that overexpressed GADD45B. A Cox multivariate analysis revealed GADD45B expression and TNM stage as significant factors affecting survival. GADD45B overexpression in patients and stage III patients were significantly associated with a poorer DFS (*P*<0.05). Therefore, we postulated that abnormal expression of GADD45B might be involved with the carcinogenesis of CRC and it showed potential for use as a prognostic marker in future.

Until now, the GADD45 gene family, including GADD45A, GADD45B and GADD45G, has been recognized as stress sensors that modulate the response of mammalian cells to genotoxic and physiological stresses and the process of tumor formation and progression [4,20-22]. GADD45B shares the common functions of the GADD45 family, including the regulation of cell growth, cell apoptosis, cellular responses to DNA damage and anti-tumor immune responses [23-25]. The overexpression of GADD45B has been shown to inhibit cell growth in a variety of human tumor cell lines, including hepatocellular carcinoma cells, prostate cancer cells, breast cancer cells and others [26-28]. To investigate the function of GADD45B in CRC, we constructed a pEGFP-N1-GADD45B vector, synthesized SiRNA duplexes, and then respectively transfected them into three CRC cell lines. After successful transfection with high efficiency, we performed an analysis of cell apoptosis using the flow cytometric method. Compared with the control group, the cells transfected with pEGFP-N1-GADD45B had more apoptotic cells, whereas the cells transfected with Si-GADD45B had fewer apoptotic cells. We also investigated the effects of pEGFP-N1-GADD45B and Si-GADD45B on the expression of apoptosis-related proteins in CRC cells by Western blotting and found that the mitochondrial apoptotic pathway was activated by GADD45B. Therefore, as a tumor suppressor gene, GADD45B could induce apoptosis in CRC cell lines. According to the results of RT-qPCR and IHC, we presumed that GADD45B might lose its normal functions and promote carcinogenesis in CRC tissues. This inconsistency of GADD45B functions between in vitro cellular-level and in vivo tissue-level may be due to complex micro-environment in human body and the resulting abnormal gene expression. The potential mechanism needs for further exploration.

Because the colon and rectum are important digestive organs, the intestinal mucosa can be easily damaged by various factors [29-31]. The human CRC-prone disorders may contribute to the risk of CRC through the accumulation of specific products resulting from DNA damage. In our study, we found that CRC had a high expression of GADD45B, which is involved in the regulation of cell growth and apoptosis. This result suggested that CRC cells might accumulate a certain amount of DNA damage, leading to dysfunction and over-expression of GADD45B. In this scenario, under chronic genotoxic stress, the failure to appropriately repair DNA damage and control growth may contribute to colorectal carcinogenesis.

In human solid tumors, DNA damage and repair defects that lead to tumorigenesis usually involve multiple factors. Defects in functional p53, p21 (*Cdkn1a*), or growth suppression genes, such as those of the GADD45 gene family, could be involved [32]. p53 is the most commonly mutated gene in cancer, exerting its activity by binding DNA response elements and regulating the transcription of specific genes in response to various stimuli, thus directing cells toward cell cycle arrest or apoptosis [33]. Because p53 functions mainly as a transcription factor, it is important to explore the genes regulated by p53 that contribute to the regulation of apoptosis. In this study, we investigated how the changes of GADD45B expression influence apoptosis-related proteins expression. Our results showed that transfection with pEGFP-N1-GADD45B in SW480 led to an increase in P53, Bax and cleavage of PARP, as well as a decrease in Bcl-2 and unactivated caspase-3. We postulated that GADD45B-induced apoptosis may be associated with the p53-Bax mitochondrial apoptotic pathway. As apoptotic pathways are frequently altered in tumor progression, proteins associated with this pathway may have potential as prognostic biomarkers. Like other researches, we have shown pro-survival function of Bcl-2, pro-apoptotic function of Bax, and elevated expression of P53 and caspase 3 in CRC tissues and ANCT [34-36]. Determination of the tumors ability to undergo apoptosis holds immense potential as a new prognostic marker for CRC.

Cancer can be viewed as the result of a succession of genetic changes during which a normal cell is transformed into a malignant one. Apoptosis is intimately connected with the elimination of potentially malignant cells, hyperplasia and tumor progression [37]. The abundance of literature suggests that defects along apoptotic pathways play a crucial role in carcinogenesis. GADD45B, as a tumor suppressor potentially through the p53-mediated apoptotic pathways, is paradoxically overexpressed in CRC and as such may play an unappreciated role in tumorigenesis. Defects in this gene might have broken some kind of balance of pro-apoptotic and anti-apoptotic proteins contributing to evasion of apoptosis and carcinogenesis.

Several studies have reported that GADD45B was decreased in human hepatocellular carcinoma (HCC) [8,38]. Compared with the high levels of staining in colon cancer, breast cancer, prostate cancer, lymphoma, squamous cell carcinoma, and leiomyosarcoma, the under-expression of GADD45B was specific to liver cancer. As a novel pituitary tumor suppressor, microarray data showed the loss of GADD45B in gonadotrope tumors where its repression modulates cell proliferation, survival, and tumorigenicity [6]. The hierarchical clustering of 19 pancreatic neuroendocrine tumors (PNETs) revealed that GADD45B was one of the most highly up-regulated genes in the malignant group of PNETs [39]. Therefore, there is still controversy regarding the expression and function of GADD45B in various tumors. Abnormal expression of GADD45B is similar to tumor suppressor p53 and phosphatase and tensin homologue (PTEN), both of which showing high expression in some head and neck cancers, and mammary cancer [40-42]. Further investigations are needed to determine the underlying mechanisms behind the dysfunction of GADD45B and CRC tumorigenesis.

## Conclusion

In this study, GADD45B was significantly overexpressed in CRC compared with ANCT. Our results suggest that increased expression of GADD45B may play an important role in CRC carcinogenesis and be used as a useful prognostic marker in future. Expression of GADD45B in CRC was abnormal; this gene may lose its normal functions as a tumor suppressor gene. We postulate that GADD45B may have undergone mutation, promotor hyper-methylation of the CpG islands or other molecular biology changes which need further investigation.

## Abbreviations

CRC: Colorectal carcinoma; ANCT: Adjacent noncancerous tissues; GADD45B: The growth arrest DNA damage-inducible, beta; HCC: Hepatocellular carcinoma; PNET: Pancreatic neuroendocrine tumors; PTEN: Phosphatase and tensin homologue.

## Competing interests

The authors declare that they have no competing interests.

## Authors’ contributions

XZ and XD conceived and designed the study. LW and XX performed the experiments. LW, XX, and DL analyzed the data. YC, YW, PW, SN and CT contributed reagents, materials, and analysis tools. LW and XX wrote the paper. All authors read and approved the final manuscript.
